# Impact of the precursor chemistry and process conditions on the cell-to-cell variability in 1T-1R based HfO_2_ RRAM devices

**DOI:** 10.1038/s41598-018-29548-7

**Published:** 2018-07-24

**Authors:** Alessandro Grossi, Eduardo Perez, Cristian Zambelli, Piero Olivo, Enrique Miranda, Robin Roelofs, Jacob Woodruff, Petri Raisanen, Wei Li, Michael Givens, Ioan Costina, Markus Andreas Schubert, Christian Wenger

**Affiliations:** 1 Dipartimento di Ingegneria, Università degli Studi di Ferrara, Via Saragat 1, Ferrara, Italy; 2 0000 0001 0142 678 IHP, Im Technologiepark 25, Frankfurt (Oder), 15236 Germany; 3 Departament d’Enginyeria Electrónica, Universitat Autónoma de Barcelona, Campus UAB, Cerdanyola del Valles, Barcelona, Spain; 4 ASM, Kapeldreef 75, Leuven, 3001 Belgium; 5 ASM America, E University Dr, 3440 Phoenix, United States; 6 Brandenburg Medical School Theodor Fontane, Neuruppin, 16816 Germany

## Abstract

The Resistive RAM (RRAM) technology is currently in a level of maturity that calls for its integration into CMOS compatible memory arrays. This CMOS integration requires a perfect understanding of the cells performance and reliability in relation to the deposition processes used for their manufacturing. In this paper, the impact of the precursor chemistries and process conditions on the performance of HfO_2_ based memristive cells is studied. An extensive characterization of HfO_2_ based 1T1R cells, a comparison of the cell-to-cell variability, and reliability study is performed. The cells’ behaviors during forming, set, and reset operations are monitored in order to relate their features to conductive filament properties and process-induced variability of the switching parameters. The modeling of the high resistance state (HRS) is performed by applying the Quantum-Point Contact model to assess the link between the deposition condition and the precursor chemistry with the resulting physical cells characteristics.

## Introduction

Resistive Random Access Memories (RRAM) gathered increasing interest in the last years^1,2^. However, an extensive research activity still has to be performed in order to improve the reliability and the switching performance at the level of RRAM array integration. The RRAM behavior is based on the electrically modification of the conductance of a Metal-Insulator-Metal (MIM) stack: the set operation drives the cell in a low resistive state (LRS), whereas the reset process switches the cell back to a high resistive state (HRS)^3–7^. The set and reset voltages with different polarities are applied to switch the cells between the HRS and LRS states. The ratio between LRS and HRS is defined as Resistance Ratio (RR). To activate the resistive switching behavior, most of the RRAM technologies require an additional preliminary forming operation^8–10^.

The choice of an optimized RRAM technology process flow providing good cell-to-cell uniformity and low switching voltages is a key issue for reliable electrical operations^11–13^. In this work, a comparison of different HfO_2_ Atomic Layer Deposition (ALD) process conditions in terms of cell-to-cell variability and reliability is performed. The deposition of the HfO_2_ films in the 1T-1R cell structures occurs at a temperature within the thermal budget of the CMOS process (*T*_*dep*_ < 400 °C), leading to the presence of hydrogen-, nitrogen-, and carbon-based residuals caused by the specific nature of the used precursors^14^. Among them, carbon atoms seem to play a major detrimental role^15^. These residuals typically act as trap levels positioned 0.8 eV below the HfO_2_ conduction band edge, impacting the switching properties of the MIM stack, consequently altering the performance and reliability of the 1T-1R cell. Carbon atoms can also cause the creation of undesired leakage paths^15^. In the case of high carbon concentrations in HfO_2_ films, similar failure phenomena can be observed after set/reset operations. Additional defects are typically generated by stress-induced thermally activated processes^16,17^, causing an increase of the conductive filament radius and the creation of parallel conductive paths, finally leading to set/reset failures.

In this paper, the switching behavior of cells during the forming, set and reset procedures is monitored by an incremental pulse and verify algorithm^18,19^. In order to analyze the peculiarity of the switching behavior activation and the process-induced inter-cell variability, 100 cells per process variation have been considered. To evaluate the endurance properties, 100 switching cycles have been performed to analyze the impact on the switching voltages on the RR. Modeling of the HRS obtained has been performed through the Quantum-Point Contact (QPC) model^20–23^ to link the technology process characteristics^24^ with cells performance and reliability.

## Results/Discussion

In order to evaluate the impact of the HfO_2_ deposition parameters on the switching characteristics of memristive devices, a halide (HA) Hf precursor was used at two different deposition temperatures (150 °C and 300 °C) and compared with a metalorganic (MO) Hf precursor used at the same deposition temperatures. The physical analysis as X-Ray diffraction (XRD) and X-ray photoelectron spectroscopy (XPS) of the HfO_2_ films were performed after finalizing the complete CMOS process flow. It should be mentioned, that the HfO_2_ films were subsequently covered by a stacked Ti/TiN layer and finally annealed at 400 °C in N_2_/H_2_ ambient for 30 min.

**Figure 1:**
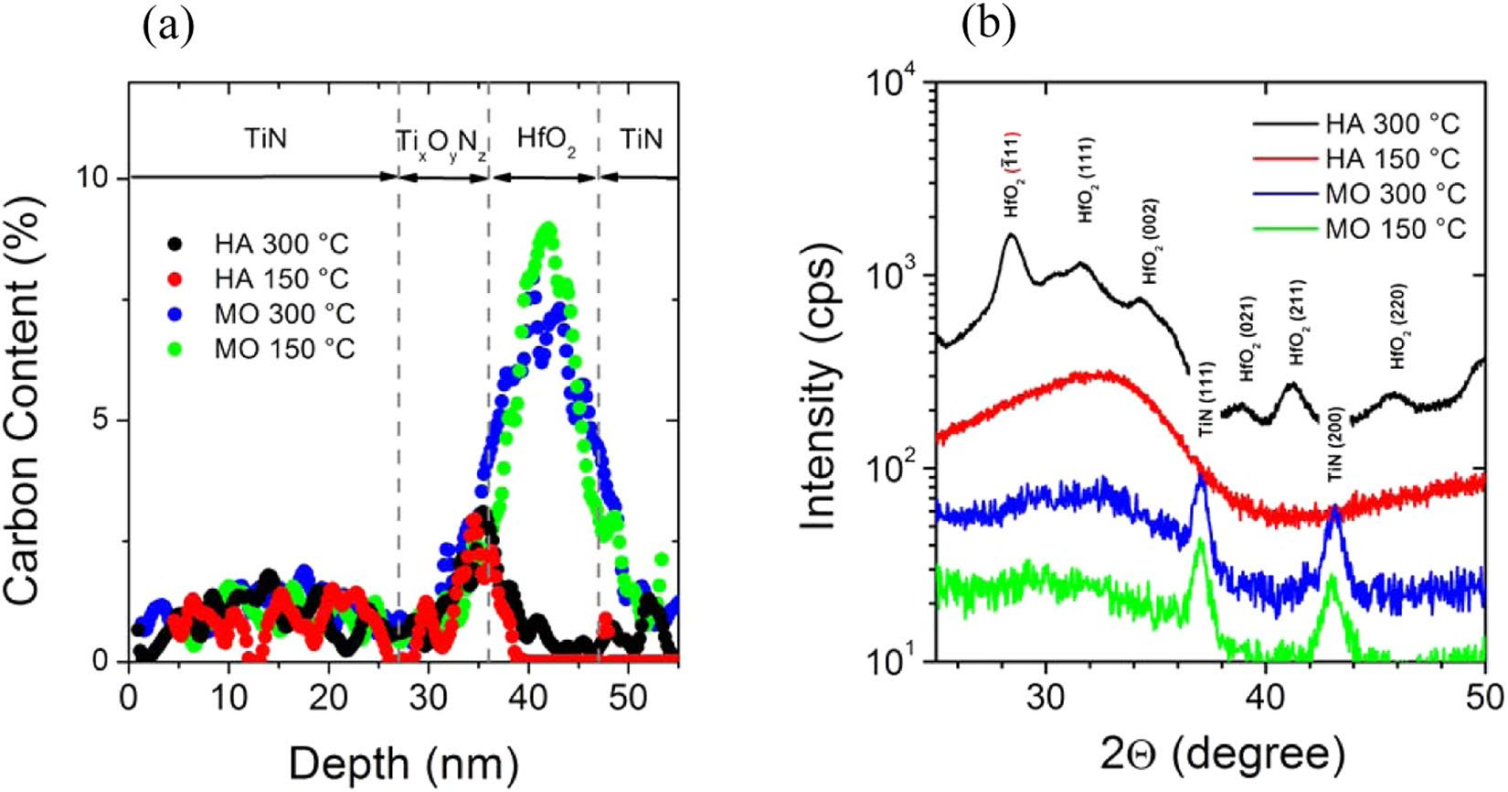
XPS (**a**) and XRD (**b**) analysis of the HfO_2_ films, deposited by ALD with different growth conditions.

**Figure 2:**
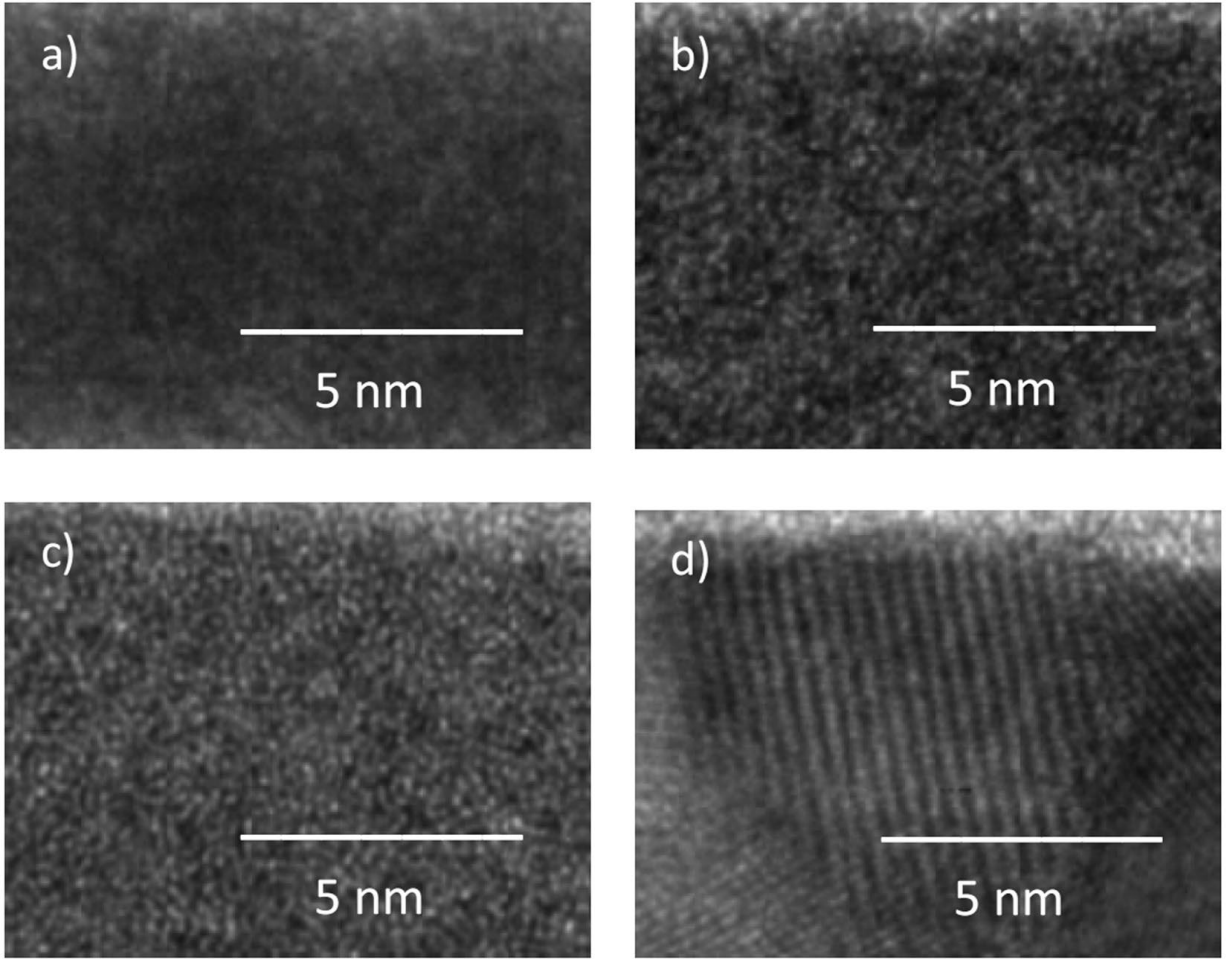
TEM images of the ALD HfO_2_ films grown by the use of MO precursor at 150 °C (**a**) and 300 °C (**b**) and by the use of HA precursor at 150 °C (**c**) and 300 °C (**d**).

As shown in Fig. 1(b), X-Ray diffraction studies were performed to study the microstructure of the HfO_2_ films. According to XRD, the HfO_2_ deposited by the use of the metalorganic precursor (MO) at 150 °C and 300 °C were grown in the amorphous state. The amorphous microstructure of thin HfO_2_ films, grown by the use of metalorganic precursor is consistent with reported ALD studies, the as-deposited amorphous films starts to crystallize after post-annealing at about 500 °C^25,26^. In contrast to the metalorganic precursor based deposition, the HfO_2_ films deposited at 300 °C by the use of the halide precursor are polycrystalline in the monoclinic phase. At the deposition temperature of 150 °C, the HfO_2_ film was grown in the amorphous state, which remains stable at post-annealing temperatures of 400 °C^27^. The crystallinity of the HfO_2_ films was further examined microscopically by Transmission electron microscopy and the results are shown in Fig. 2. The HfO_2_ films, grown by the use of the MO precursor are amorphous (Fig. 2(a,b)), while the HfO_2_ film, deposited using the HA precursor at 300 °C is grown in a polycrystalline structure. However, the obtained TEM results confirm the previous X-Ray studies.

The carbon content, oxygen concentration and Hf/O ratio in the HfO_2_ films are analyzed by depth profiling of the memristive stack by using X-ray photoelectron spectroscopy. As illustrated in Fig. 1(a), the carbon content in the HfO_2_ films is strongly affected by the precursor. Using the halide precursor strongly reduces the carbon content in the HfO_2_ to less than 1%, while the films deposited by the metalorganic precursor contain 7–9% carbon residuals caused the molecular structure of the used metalorganic precursor^25^. It has to be added, that the small carbon peak at the interface between HfO_2_ and the Ti_x_O_y_N_z_ film is caused by the vacuum break between the HfO_2_ ALD and the growth of the Titanium layer by PVD (Plasma Vapor Deposition). In addition, the oxygen content is found decreasing with increasing amount of carbon atoms, as listed in Table 1. The different process conditions were labelled as A, B, C and D as summarized in Table 1.

**Table 1:** Process Description.

Proc.	Precursor	T_dep_ (°C)	Carbon content	Oxygen content	Microstructure
A	HA	300	0.3%	58%	poly-crystalline
B	HA	150	0.4%	56%	amorphous
C	MO	300	7%	49%	amorphous
D	MO	150	9%	41%	amorphous

During the CMOS fabrication flow of the RRAM array, several annealing steps at 400 °C for 30 min were applied. These steps activate the scavenging properties of the Ti layer, resulting in the oxygen content reduction in the HfO_2_ layer^28^. Due to the layer asymmetry of the resistive MIM device, the distribution of the oxygen vacancies in the HfO_2_ layer is not uniform. There is a strong gradient from the top interface (i.e., with Ti) to the bottom interface (i.e., with TiN) of the HfO_2_ film. Hence the sensitivity of the XPS depth profile setup is not sufficient to provide the exact oxygen vacancies content but sufficient to provide the oxygen concentration. In case of carbon, the concentration can be assumed as homogenous since the carbon atoms are incorporated through the deposition process and not during the annealing step.

**Figure 3:**
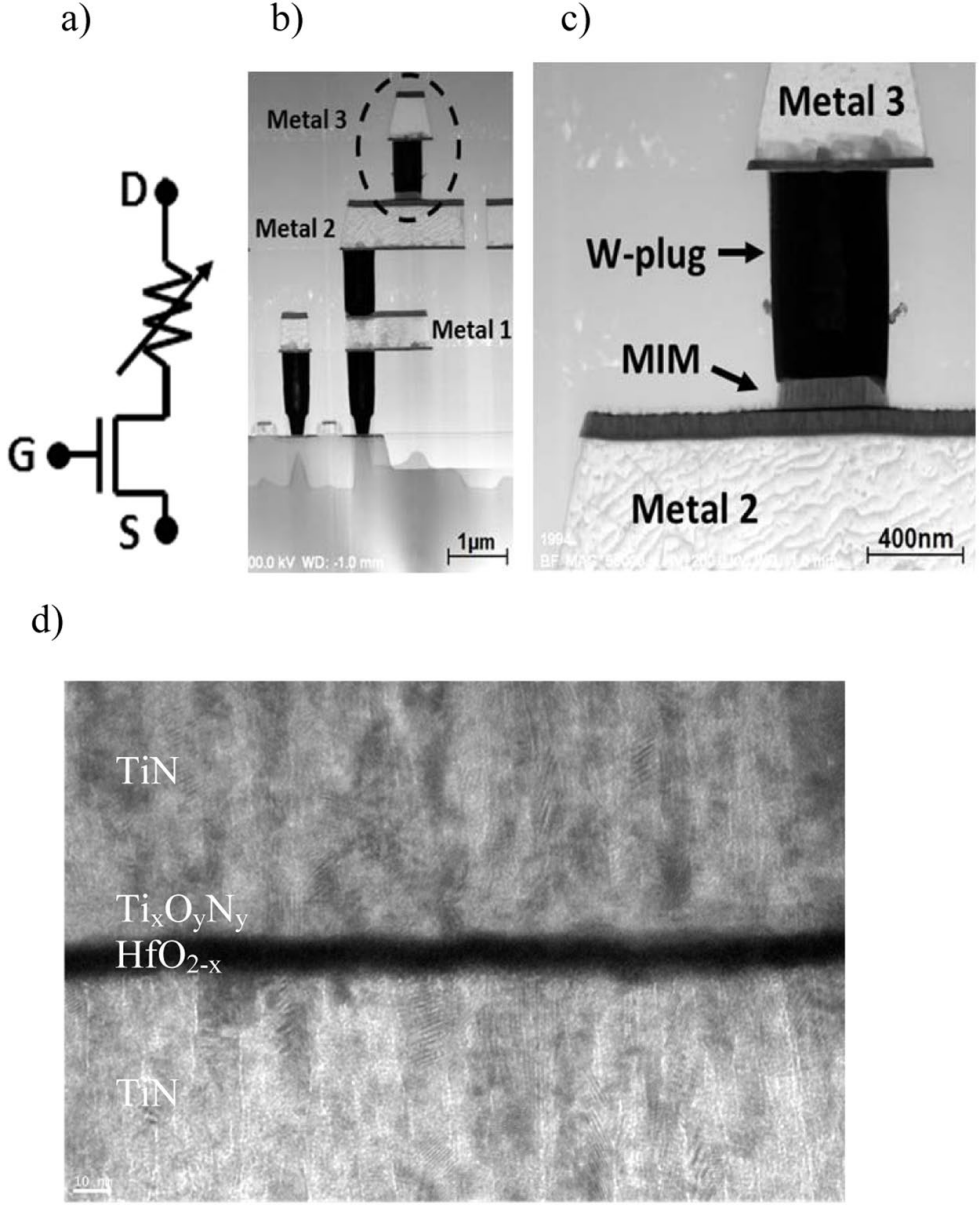
Schematic diagram (**a**), cross-sectional TEM image of the 1T-1R cell (**b**), MIM stack insight (**c**), and high magnification TEM image of the memristive MIM device (**d**).

The schematic and cross-sectional TEM images of the integrated RRAM cell including the metal lines, the MIM materials and the Tungsten-based via connections are shown in Fig. 3.

**Figure 4:**
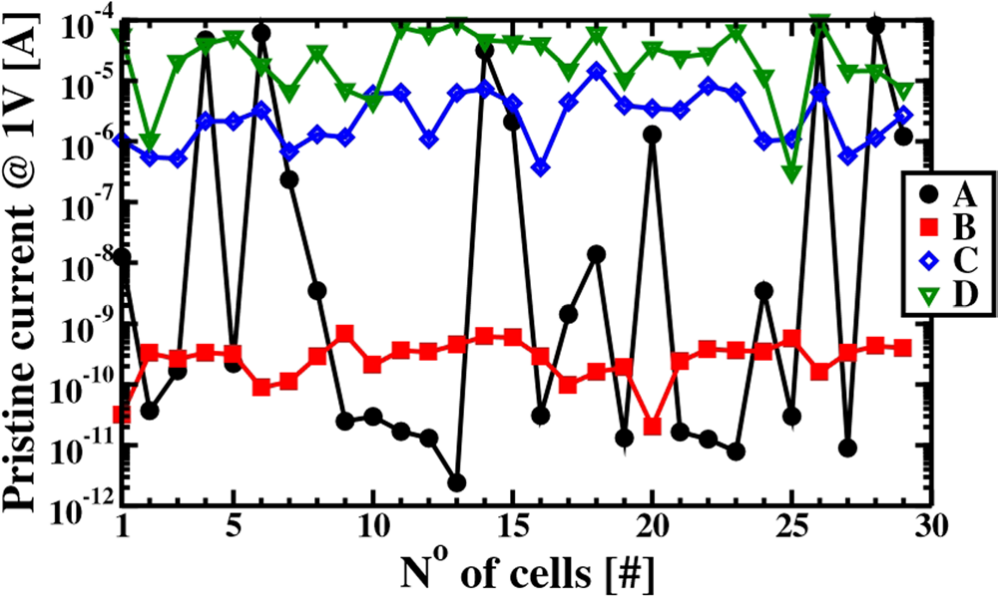
Pristine currents measured at 1 V of the four deposition conditions after CMOS fabrication, including the Ti-scavenging activation step at 400 °C.

In order to study the impact of the HfO_2_ deposition condition on the switching behavior systematically, the pristine currents of the memristive devices at 1 Volt are investigated primarily. As shown in Fig. 4, the currents are strongly affected by the deposition conditions as well as the gettering activities of the Ti layers. In case of the amorphous microstructures of HfO_2_: with increasing carbon content, the current is also increasing. The large pristine current fluctuations between different cells of the poly-crystalline HfO_2_ film (process A) are caused by the high affinity of the grain boundaries to charged oxygen vacancies causing leakage paths in some of the cells^29,30^.

Afterwards, the memristive cells were formed by the so-called form and verify algorithm. The cumulative distributions of the forming voltages are shown in Fig. 4(a). The films deposited by the process conditions C and D are the ones where the pristine currents (see Fig. 3) are the largest caused by the high carbon content and the low oxygen concentration in the HfO_2_ layers. Lower electric potentials are needed to form these memristive cells.

**Figure 5:**
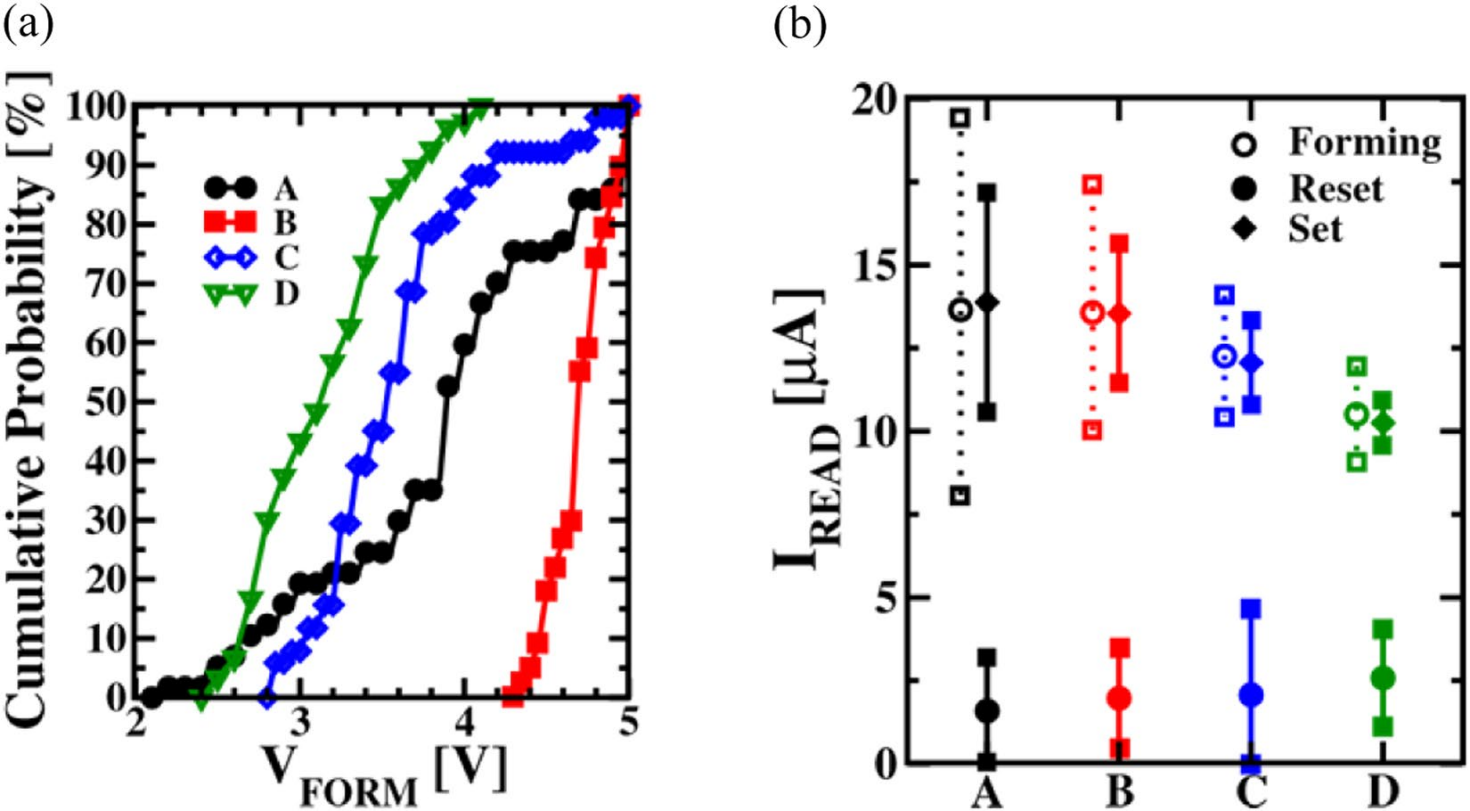
Cumulative distribution of the Forming voltages measured as function of the deposition condition A, B, C and D (**a**). Average read-out current values and standard deviations after forming, set and reset (**b**).

Process B features a very low carbon concentration in the amorphous dielectric layer, resulting in a slightly difficult forming operation (i.e., larger voltage requested as shown in Fig. 5(a)), but with a higher degree of uniformity as shown by its pristine current. The films deposited by process A exhibit a very low carbon concentration, although the poly-crystalline structure of the dielectrics makes it less reliable and controllable in terms of forming, as shown in Fig. 5(a). All processes with amorphous microstructures are providing a clear trend: Larger carbon concentration increase the pristine currents, causing a decrease of the median forming voltage, a decrease of the median read current after forming/set, and an increase of the median read current after reset. The decrease of the LRS currents with raising carbon content, as illustrated in Fig. 5(b) could be related to the higher probability of having carbon atoms next to the narrowest part of the filament. Such atoms could create preferential conductive paths, repelling the oxygen vacancies from moving into that region and limiting the conductive filament growth^22^.

**Figure 6:**
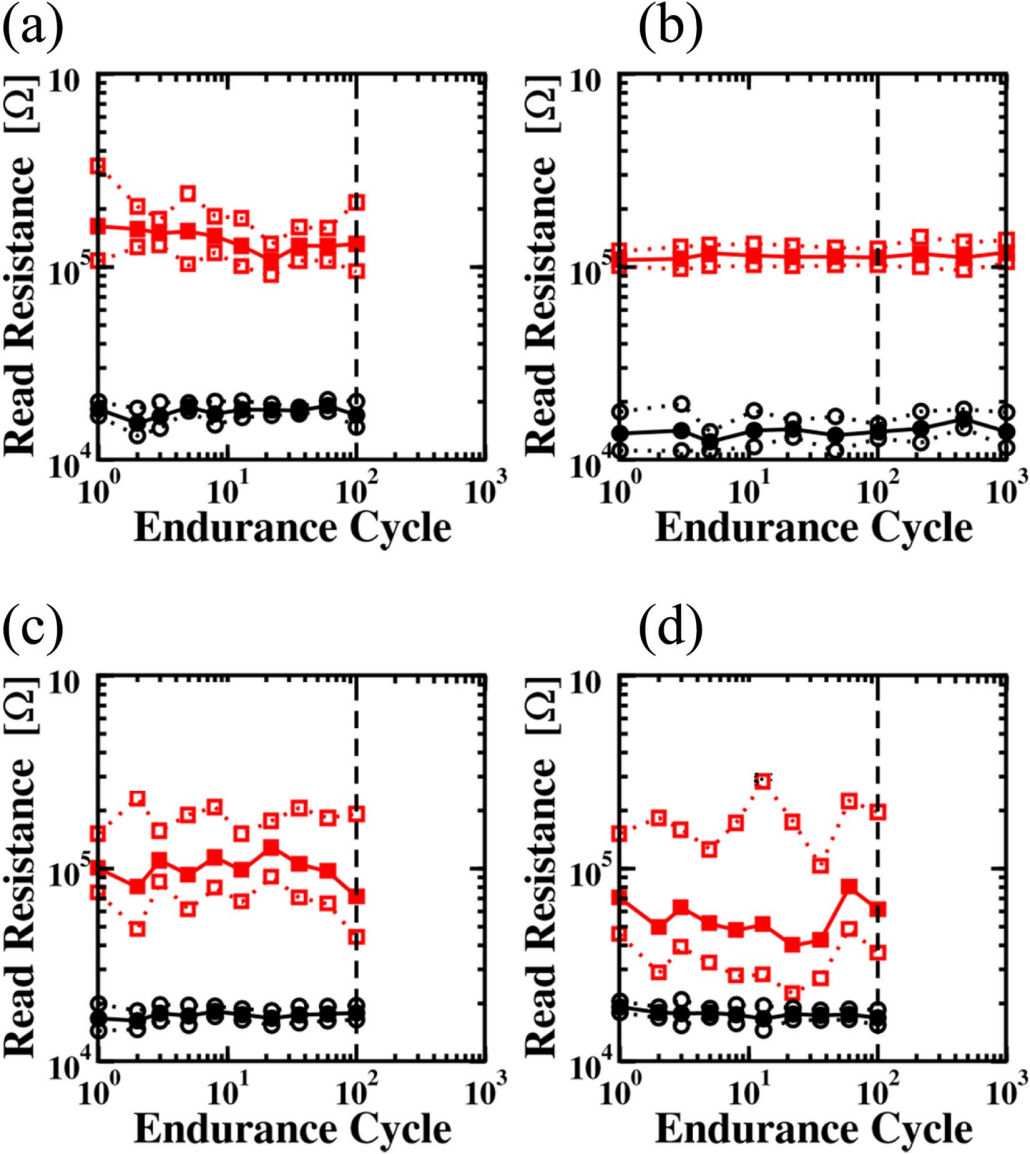
Average HRS and LRS resistances and their standard deviation evaluated from 20 memristive cells deposited by the processes A (**a**), B (**b**), C (**c**) and D (**d**) as function of resistive cycling. The average values of the resistance values are illustrated by the solid symbols (●for LRS and  for HRS), while the open symbols (○, ) represent the standard derivations.

In order to identify the endurance characteristics of the memristive devices, 100 to 1000 cycles using the incremental pulse and verify algorithm were performed. The average HRS and LRS resistances and their standard deviation were evaluated by cycling 20 cells per deposition process variation. As illustrated in Fig. 6, the HRS and LRS states of process B remain stable during the endurance cycling, mainly due to the low content of carbon as well as the amorphous microstructure of the HfO_2_ film. With increasing carbon content (processes C and D) or introducing grain boundaries (process A) the stability and controllability of the HRS states is reduced due to an increase of the leakage current, causing a reduction of the resistance ratio.

**Figure 7:**
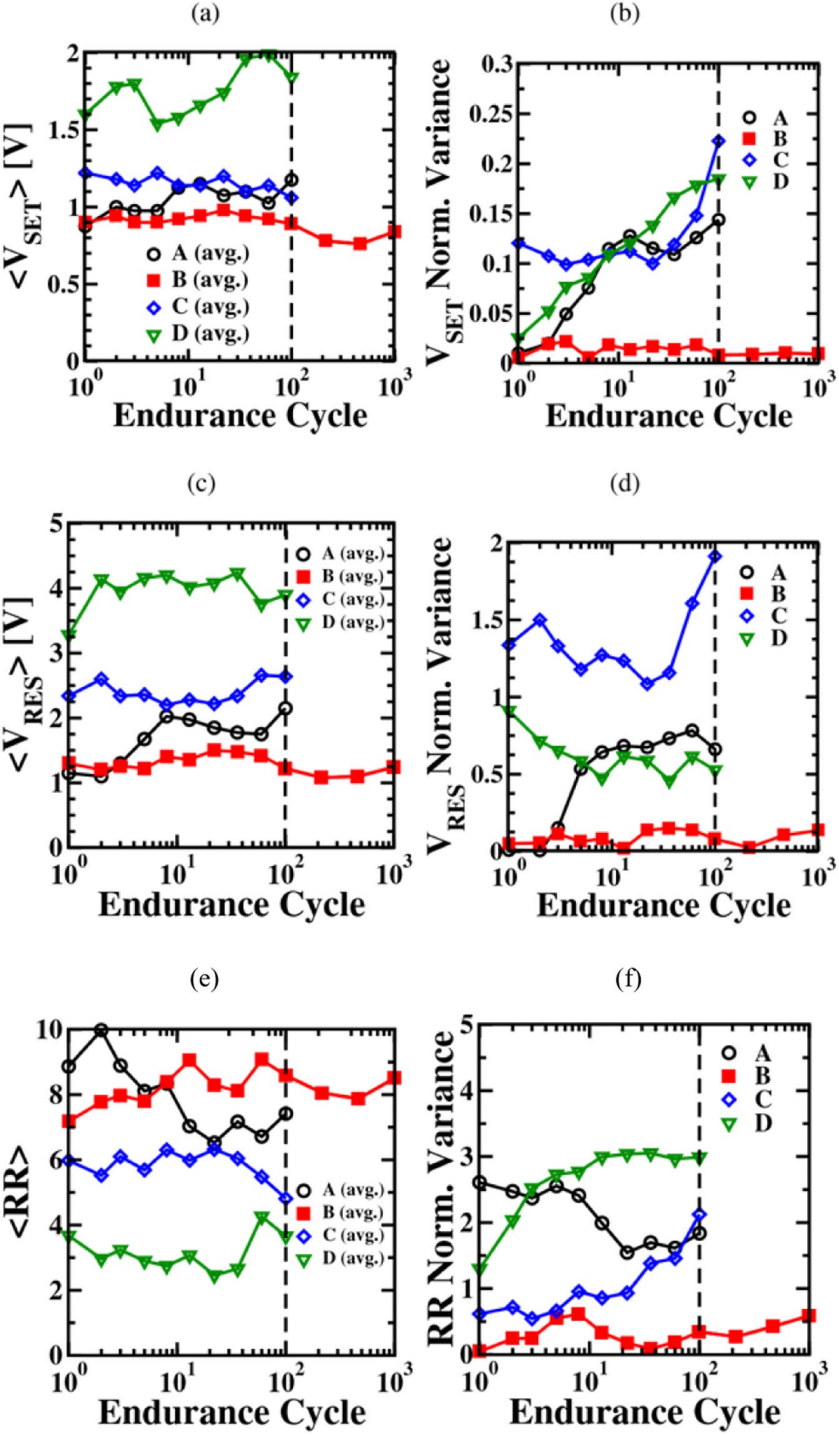
Mean V_SET_ (**a**) and V_RES_ (**c**) values and their normalized variances (**b**), (**d**) evaluated from the IV characteristics of 20 memristive cells processed by the variation A-D as function of programming cycles. The evolution of the average resistance ratio values RR and the normalized variance of RR as function of cycles are illustrated in (**e**) and (**f**).

The resistance ratio (RR), V_SET_, and V_RES_ average values and normalized variances (i.e., the ratio between variance and the average value) as function of cycling are reported in Fig. 7. The Halide based precursor processes (A, B) reach the thresholds of the set and reset algorithms at lower V_SET_/V_RES_ values with respect to the metalorganic precursor based processes (C, D). As shown in Fig. 7(a), process A demonstrates the largest RR at the beginning of the cycling stress, but also a fast reduction during the endurance test. A better stability of the RR during cycling is observed for the amorphous HfO_2_ films. Moreover, the endurance performance seems to be related to the carbon content: film B, corresponding to the amorphous HfO_2_ layer with the lowest carbon content, demonstrates the highest RR with the lowest normalized variance (see in Fig. 7(b)) after 100 cycles with an excellent stability during cycling. There is a clear trend: With increasing carbon content, RR is reduced.

As shown in Fig. 7(c,e), the V_SET_ and V_RES_ values increase with raising carbon content in the amorphous films. Considering the variances, illustrated in Fig. 7(d,f), devices B still demonstrate the lowest values and the highest stability during cycling, confirming that the carbon content plays a fundamental role on cells’ performance and reliability^21^. This means, when the conductive filaments in the devices B are correctly formed, the subsequent set and reset operations are not impacted by carbon impurities, resulting in reduced dispersion of the switching voltage values. The cycling behavior of the memristive devices C and D is different, the subsequent set/reset operations are more difficult and the required voltages for set/reset switching increase as well as their dispersion. Moreover, the oxygen concentration, which decreases when the carbon content increases, plays a role. The reduced concentration of oxygen vacancies reduces the ion mobility^31^, hence a higher voltage is needed to move the oxygen vacancies in order to recreate and rupture the filament. Since the deposition process B provides the best performance of the memristive devices after 100 cycles, the endurance test has been extended to 1000 cycles: no relevant variation of the parameters has been observed.

In order to correlate the obtained experimental results, obtained by pulsed induced switching, with the quantum mechanical nature of the currents in LRS and HRS, additional DC measurements were performed. To understand the impact of the carbon within the HfO_2_ film on the cells’ conduction properties after the reset process, the quantum point contact model is applied to the IV characteristics. In this regard, the Quantum Point Contact (QPC) model has been used^16^, allowing the interpreation of the I-V characteristics measured after the reset operation by the following equation:

1 $${I}_{HRS}=\frac{2e}{h}G/{G}_{0}(eV+\frac{1}{\alpha }ln[\frac{1+{e}^{\alpha (\varphi -\beta eV)}}{1+{e}^{\alpha [\varphi +(1-\beta )eV]}}])$$

The model parameters are the barrier height Φ which is associated with the bottom of the first quantized level, the curvature parameter α, which is related to the potential barrier curvature, assuming a parabolic longitudinal potential and the symmetry parameter β which represents the fraction of the applied bias that drops at the source side of the conductive filament and defines the constriction symmetry. When β is very close to 1, almost the complete applied bias voltage drops at the source side of the filament, hence the constriction is highly asymmetric. The quantum conductance unit G_0_ = 2e^2^/h corresponds to the conductance of a single conduction mode nanowire, where e is the electron charge and h the Planck’s constant. The parameter G/G_0_ represent the non-ideality of the filaments. This parameter should be 1 in case of an ideal filament structure. The series transistor integrated in the memristive 1T-1R cells (see Fig. 2) has a significantly large area with a proper current driving capability (i.e., W = 1.14 µm, L = 0.24 µm, µ_0_ = 1000 cm^2^/Vs, t_ox_ = 5 nm). During the read operation the transistor constantly works in the linear region with a fixed resistance which is negligible compared to one of the 1 R element. Therefore, the transistor is not included in the simulation of the HRS current by the QPC model.

**Figure 8:**
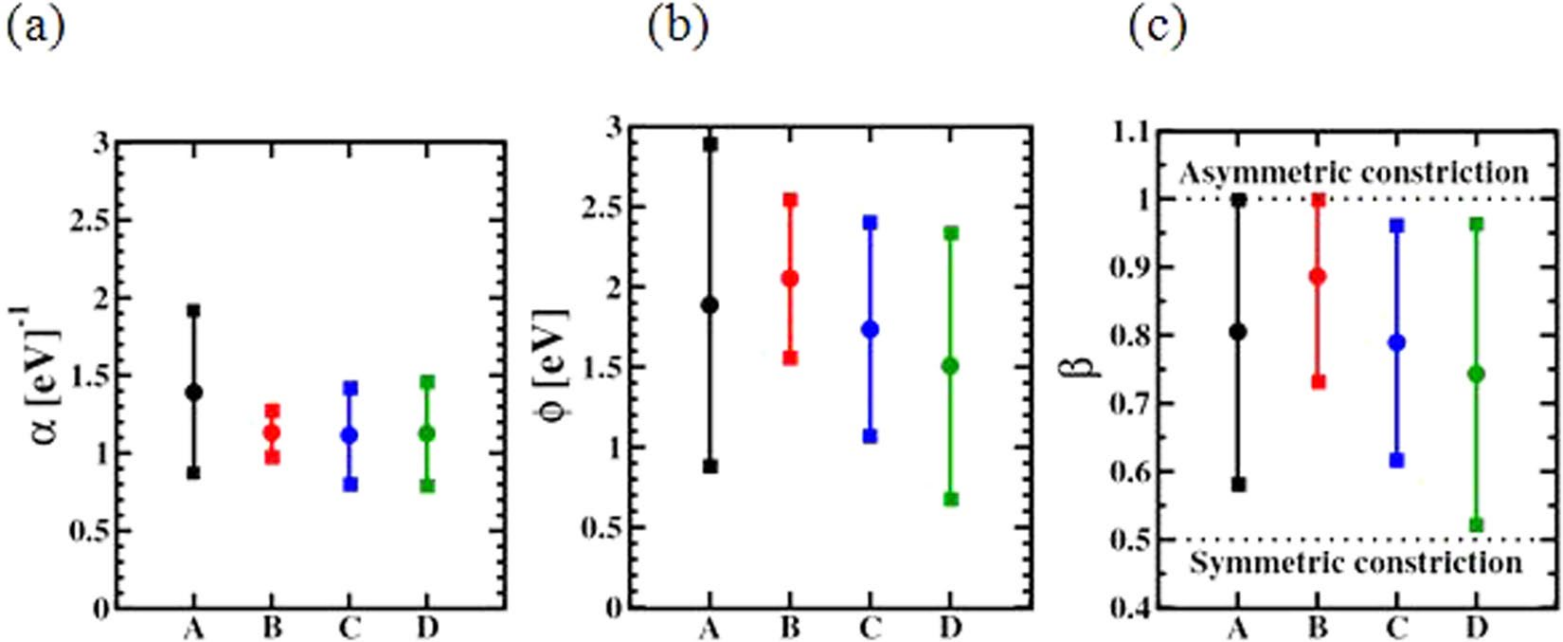
Extracted QPC parameters α (**a**), Φ (**b**) and β (**c**) as function of the depositon process for the growth of HfO_2_ films.

The average values and standard deviations of the fitting parameters are illustrated in Fig. 8. The extracted curvature parameter *α* is quite similar for the amorphous HfO_2_ films grown by the process conditions B, C and D, while *α* is slightly larger in the poly-crystalline film HfO_2,_ deposited by process A. Larger *α* could be interpreted as an increase of the width of quantum mechanical barrier. This increasement could be ascribed to the change of the shape of the constriction caused by the microstructure of the HfO_2_ film. The width of the barrier and its variability is mainly impacted by the microstructure of the HfO_2_ film and is not affected by the carbon content.

Concerning the impact of the carbon content to the height of the quantum mechanical barrier, Φ is decreasing with raising the carbon content, as illustrated in Fig. 8(b). The decreasing barrier height leads to larger HRS currents, as illustrated in Fig. 6(b) and consequently to a reduction of the resistant ratio, as shown in Fig. 7(e).

Within the series of amorphous HfO_2_ films, the process variation B leads to the lowest variability of the barrier height Φ, providing the highest cell-to-cell uniformity. The largest variability of the barrier height is evaluated for HfO_2_ films deposited by process A, which is mainly caused by the poly-crystalline structure.

**Figure 9:**
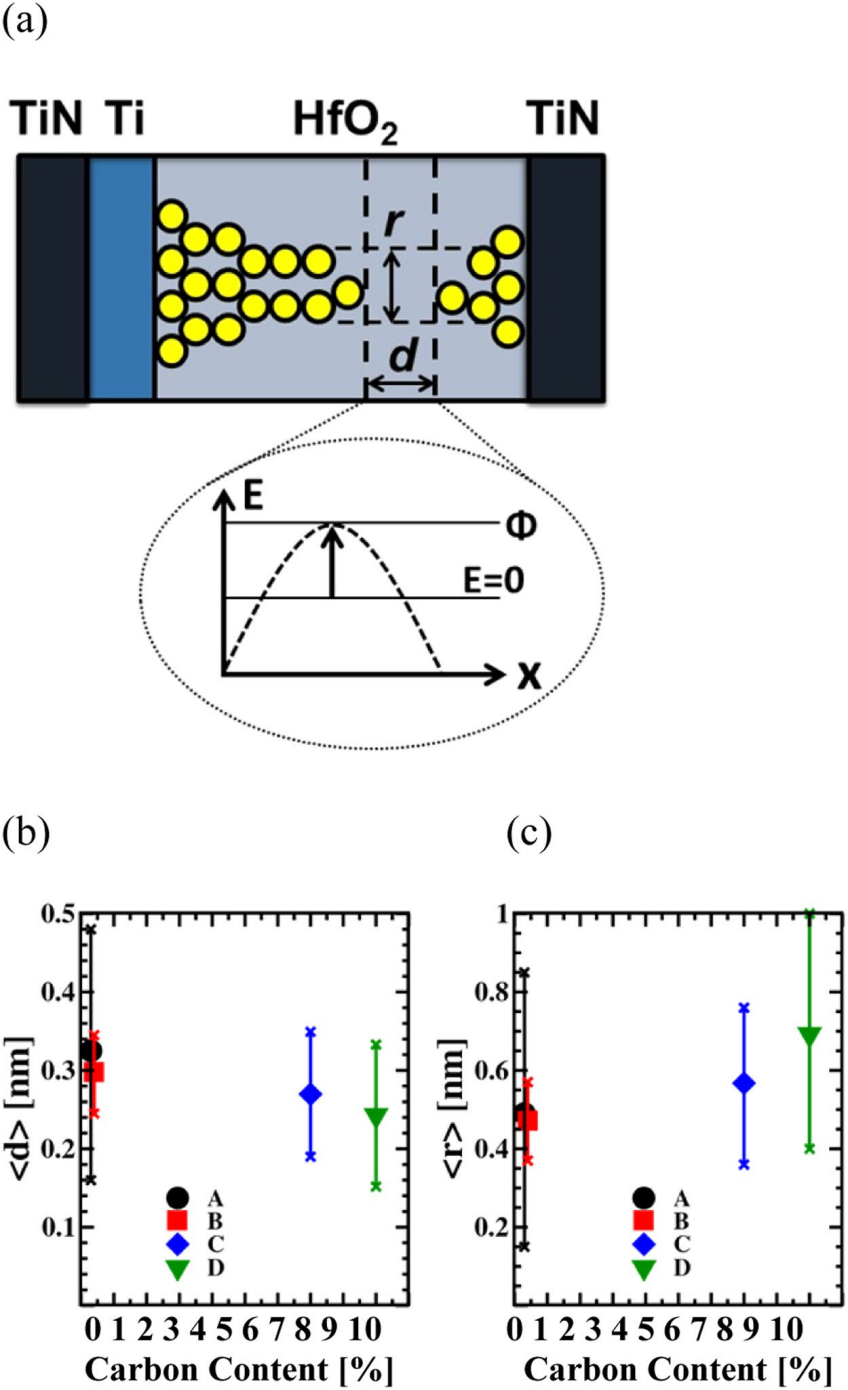
Schematic illustartion of the conductive filament shape after the reset procedure (**a**). Average values of calculated barrier length *d* (**b**) and radius of the filament constriction *r* (**c**). The error bars indicate the standard deviation.

The QPC parameter β is defining the position of the constrction point. Independent of the process conditions, the evaluated values for the parameter β are close to 1, as illustrated in Fig. 8(c). Therefore the constriction point is located next to the bottom electrode of the MIM cell. The position of the constriction point is mainly impacted by the architecture and the process flow of the MIM cell as shown in Fig. 9(a), creating an asymmetric constriction due to the presence of the Ti layer^18^. However, the presence of carbon residuals as well as the microstructure of the HfO_2_ layer don’t have an impact to the asymmetric position of the constriction point.

In addition to the extracted quantum mechanical parameters, the relationship between α and the potential barrier thickness d can be calculated as^16,32^:

2 $$d=\frac{h\alpha \sqrt{\varphi }}{{\pi }^{2}\sqrt{2{m}^{\ast }}}$$

where m^*^ is the electron effective mass in the constriction. The equivalent radius r of the constriction point, corresponding either to a single filament or to multiple conductive filaments in parallel, can be calculated as:

3 $$r=h{z}_{0}/2\pi \sqrt{2{m}^{\ast }\varphi }$$

where z_0_ = 2.404 is the first zero of the Bessel function J_0_^16^. The HRS structure of the filament obtained after the reset process is sketched in Fig. 9a).

The average values of d and r as a function of the carbon content are illustrated in Fig. 9(b,c). The average barrier width is decreasing with increasing carbon content, whereas the average radius is increasing.

The impact of carbon residuals on the width of the barrier d and the conductive filament constriction radius r could be explained by additional trap levels inside the HfO_2_ band gap formed by carbon ions^21^, generating a reduction of the barrier.

When the carbon content increases, the LRS current decreases, as illustrated in Fig. 5(b). This negative impact could be related to the creation of partially formed filaments involving carbon defects, which prevent the complete growth of the oxygen vacancy based conductive filament growth. To sum up, the origin of the cell-to-cell variability can be enlightened by the use of the QPC model. By applying the QPC model to the DC-switching curves, the variability is affected by he microstructure of the deposited dielectric film as well as its carbon content.

## Conclusion

The impact of the precursor chemistry as well as the affect of the deposition temperature have been studied. In order to evalaute the endurance characteristics of the memristive devices, 100 switching cycles using the incremental pulse and verify algorithm were performed. With increasing carbon content or introducing grain boundaries in the HfO_2_ films, the stability and controllability of the HRS states is reduced due to an increase of the leakage current, causing a reduction of the resistance ratio. The grain boundaries of the poly-crystalline HfO_2_ films are causing a high cell-to-cell variability during the endurance test.

Amorphous HfO_2_ films deposited by using the halide precursor provide the highest inter-cell and intra-cell uniformity. Metalorganic precursors-based processes result in amorphous HfO_2_ films as well, although the carbon content is higher. The inter-cell uniformity seems to be affected by the carbon content: HfO_2_ films with high carbon content show reduced restistance ratios and an increased variability of the set and reset parameters.

In order to understand the impact of the carbon content in the HfO_2_ films on the cells’ switching characteristics, the quantum point contact model was applied to the IV curves. The height of the quantum mechanical barrier is decreasing with raising carbon content. The decreasing barrier height leads to larger HRS currents and consequently to a reduction of the resistant ratio. In contrast to the height of the barrier, the width of the barrier and its variability is mainly impacted by the microstructure of the HfO_2_ film and is not affected by the carbon content.

In conclusion, HfO_2_ based memristive cells manufactured with halide precursors at low deposition temperature provide the most promising results in terms of cell-to-cell variability and switching reliability.

## Methods

### Preparation and analytical characterization of the HfO_2_ based 1 T1R RRAM arrays

The 1T-1R memory cells are constituted by a select nMOS transistor manufactured in BiCMOS technology (width of 1.14 *µ*m and length of 0.24 *µ*m), which also sets the current compliance, whose drain is in series to a MIM stack. The MIM area is equal to 0.4 *µ*m^2^. Metal 1 as well as Metal 2 are metallic layer stacks, consisting of Ti/TiN/Al/TiN/Ti. The MIM integrated on the metal line 2 of the BiCMOS process is composed by 150 nm TiN top and bottom electrode layers deposited by magnetron sputtering, a 7 nm Ti layer, and an 8 nm HfO_2_ layer deposited through thermal ALD with the four different processes. A halide (HA) Hf precursor (HfCl_4_) was used for processes A and B, whereas for processes C and D a metalorganic (MO) Hf precursor was used in combination with H_2_O as oxygen source.

The process flow of the samples used for XRD analytics was slightly modified. The HfO_2_ films, deposited by the MO-based precursor were grown on TiN films, which were grown by atomic vapour deposition (AVD). These TiN films were deposited at 400 °C from a pure Ti(NEt_2_)_4_ precursor and NH_3_ by AVD. The HfO_2_ grown by the HA-based precuror are deposited on PVD TiN. The PVD TiN layers were reactively deposited using a d.c. magnetron sputtering of Ti and nitrogan as reactive gas at room temperature.

The carbon content, oxygen concentration and Hf/O ratio in the MIM stack are analyzed via X-ray photoelectron spectroscopy (XPS) for all processes. The XPS measurements were performed after annealing the MIM stack at 400 °C for 30 minutes. This annealing step activates the scavenging properties of the Ti layer, resulting in the oxygen content reduction in the HfO_2_ layer. Due to the layer asymmetry of the resistive MIM device, the distribution of the oxygen vacancies in the HfO_2_ is not uniform. There is a strong gradient from the top interface (i.e., with Ti) to the bottom interface (i.e., with TiN) of the HfO_2_ film, hence the sensitivity limit of the XPS depth profile is too small to provide the exact oxygen vacancies content but sufficient to provide the oxygen concentration. In case of carbon, the concentration can be assumed as homogenous since the carbon atoms are incorporated through the deposition process and not during the annealing step.

### Electrical characterization

The test environment for cells characterization consists in a Keithley 4200-SCS wafer-level tester. The Forming/Set/Reset operations were performed by using an incremental step pulse (V_STEP_ = 0.1 V) and verify algorithm^18^. A sequence of increasing voltage pulses is applied on the drain of the cell during Forming and Set, with a transistor gate voltage V_G_ = 1.5 V to set the Forming/Set current compliance, whereas the sequence of increasing voltage pulses is applied on the source of the cell during Reset, with a transistor gate voltage V_G_ = 2.8 V which leads to a 120 µA compliance current. All pulses feature duration of 10 µs in order to maximize the switching yield^7^. After every pulse a read-verify operation is performed, where the cell current I_read_ was measured by applying 0.2 V on the drain of the cell with V_G_ = 1.5 V and a read time T_read_ = 10 µs. When the read current reaches I_target_ = 10 µA the Forming and Set operations are stopped, whereas during Reset the operation is stopped when the read current reaches I_target_ = 2 µA. V_FORM_, V_SET_ and V_RES_ denote the voltages at which the algorithms targets are reached during Forming, Set and Reset operations, respectively. These parameters reflect the operation of the memory when a Set/Reset algorithm is considered, since they guarantee that a sufficiently high read margin is obtained.

In the DC mode used for QPC characterization, V_D_ was raised from 0 to 2 V during the set operation with V_G_ = 1.5 V and V_S_ was increased from 0 to 2 V during the reset procedure with fixed V_G_ = 2.8 V, respectively. A sweep ramp of 1 V/s was used.
